# *Ex vivo* isolation, expansion and bioengineering of CCR7+CD95-/or CD62L+CD45RA+ tumor infiltrating lymphocytes from acute myeloid leukemia patients’ bone marrow

**DOI:** 10.1016/j.neo.2021.11.003

**Published:** 2021-11-11

**Authors:** Huynh Cao, Do Hyun Kim, Ashley Howard, Hector Moz, Samiksha Wasnik, David J. Baylink, Chien-Shing Chen, Mark E Reeves, Saied Mirshahidi, Jeffrey Xiao, Olivia Francis, Guido Marcucci, Yi Xu

**Affiliations:** a Divisions of Hematology and Oncology, Loma Linda University, Loma Linda, United States; b Regenerative Medicine, Department of Medicine, Loma Linda University, Loma Linda, United States; c Loma Linda University Cancer Center, 11234 Anderson Street, Loma Linda, CA 92354, United States; d Department of Medicine and Basic Sciences, Biospecimen Laboratory, Loma Linda University Cancer Center, Loma Linda University School of Medicine, Loma Linda, CA 92354, United States; e Department of Pharmaceutical and Administrative Sciences, School of Pharmacy, Loma Linda University, Loma Linda, CA, United States; f Gehr Family Center for Leukemia Research, Hematology Malignancies and Stem Cell Transplantation Institute, City of Hope Medical Center, Duarte, CA, United States

**Keywords:** Acute myeloid leukemia, Tumor-Infiltrating Lymphocytes, Immunotherapy, Naïve T, CCR7, CD95, CD62L, CD45RA, Bone marrow, Adoptive cell therapy, Interleukin, Programmed cell death protein 1, PD-1, CAR-T, AML, acute myeloid leukemia, TILs, tumor-infiltrating lymphocytes, BM, bone marrow, BMMNC, bone marrow mononuclear cells, HSCs, hematopoietic stem cells, FACS, flow cytometry, IL, interleukin

## Abstract

T cell based immunotherapies can be applicable to acute myeloid leukemia (AML). Therefore, the selection of optimal T cells, cell manufacturing, and therapeutic T cell engineering are essential for the development of effective adoptive T cell therapies for AML. Autologous tumor-infiltrating lymphocytes (TILs) have been in clinical trials to treat solid malignancies. Herein, we assessed whether TILs can be isolated from the bone marrow (BM) of AML patients, expanded *ex vivo* and utilized as a novel therapeutic strategy for AML. To this end, firstly we analyzed the immunophenotypes of a series of primary BM samples from AML patients (*N* = 10) by flow cytometry. We observed a variable amount of CD3+ TILs (range ∼2.3–∼32.6% of mononuclear cells) among BM samples. We then developed a novel protocol that produced a three-log *ex vivo* expansion of TILs isolated from AML patient BM (*N* = 10) and peripheral blood (PB) (*N* = 10), including from patients with a low number of CD3+ T cells, within 3, 4 weeks. Further, we identified previously described naïve T cells (CCR7+CD95-/or CD62L+CD45RA+) in AML BM and PB samples, which seemed to be required for a successful TILs *ex vivo* expansion. Finally, we showed that the expanded TILs could: (1) cause cytotoxicity to autologous AML blasts *ex vivo* (90.6% in control without T cell treatment vs. 1.89% in experimental groups with PB derived T cells and 1.77% in experimental groups with BM derived TILs, *p* < 0.01), (2) be genetically engineered to express CYP27B1 gene, and (3) infiltrate the BM and reside in close proximity to pre-injected autologous AML blasts of engrafted immunodeficiency mice. Altogether, these results provide a rationale for further studies of the therapeutic use of TILs in AML.

## Introduction

AML is a hematological malignancy that is characterized by infiltration of the bone marrow, blood, and other tissues by poorly differentiated blasts [1]. Intensive induction chemotherapy for fit patients and subsequent allogeneic hematopoietic stem cell (HSCs) transplantation (Allo-HCT) have resulted in long-term remission 2, 3, 4. However, treatment failure is common, as manifested by disease refractory and relapse [5]. One of the major difficulties in AML treatment is the inability to track and recognize antigens found on leukemia stem cells (LSCs), and eliminate those quiescent and chemotherapy-resistant LSCs 6, 7, 8. In addition, the treatment options for older, unfit patients are limited with molecular targeted therapies offering palliation but they do not lead to disease remission. Thus, the outcome for this specific population remains dismal, with a median survival of only 5 to 10 months. Therefore, a novel, effective therapy is an unmet need for AML patients with relapsed/refractory disease and elderly patients [9,10].

Cancer immunotherapy utilizes components of the immune system to eliminate cancer cells while sparing healthy cells [11,12]. Bioengineering T cells by generating Chimeric Antigen Receptor T cells (CAR-T) is a new approach to provide precise and personalized immunotherapy for each cancer patient [13]. Albeit effective in acute lymphocytic leukemia (ALL), the applicability of CAR-T to AML remains to be fully proven. Recently, engineered tumor infiltrating lymphocytes (TILs) or marrow-infiltrating lymphocytes (MILs) have received widespread attention because of their efficacy in treating metastatic solid tumors [14] and multiple myeloma [15]. In contrast to CAR-T therapies, TILs have the advantage of being able to respond to multiple signals from the tumor microenvironment and antigens on cancer cells [16,17]. However, problems including deficiency, dysfunction and exhaustion of TILs in the cancer microenvironment remain to be solved [18,19].

We hypothesize here that TILs can be found in the bone marrow (BM) of AML patients and that derived TILs combined with immune checkpoint blockage is a novel, therapeutic strategy for AML.

## Materials and methods

The list of reagents including manufacturers and catalogues of antibodies and kits are found in the supplementary data (**Supplementary Table 1**).

### Human samples

AML BM samples (**Patients #1–10,** Table 1) were obtained from Loma Linda University Cancer Center Biospecimen Laboratory (LLUCCBL). AML Peripheral Blood and BM samples (**Patients #11–20,** Table 1) were obtained from the City of Hope National Medical Center (COHNMC). All donor patients signed an informed consent form. Sample acquisition was approved by the Institutional Review Boards at the LLUMC and the COHNMC in accordance with an assurance filed with and approved by the Department of Health and Human Services, and it met all requirements of the Declaration of Helsinki.

**Table 1:** List of AML patients for the FACS screening and *ex vivo*/*in vivo* studies.

No.	Diagnosis	Disease Status	Age	Sex	Cytogenetics(Karyotype)	Gene Mutation
1	AML	Newly Diagnosed	32	F	Normal	FLT3, CEBPA and NPM1: NEGATIVE
2	AML	Newly Diagnosed	70	M	Normal	The molecular analyses of CEBPA, FLT3and NPM1 mutations show only positive for FLT3 internal tandem duplication mutation (FLT3-ITD)
3	AML	Diagnosed	35	M	Normal	FLT3, CEBPA and NPM1: NEGATIVE
4	AML	Newly Diagnosed	59	M	Normal	CEBPA negative; NPM mutation not detected; FLT3 ITD not detectedFLT3 TKD not detected
5	AML	Newly Diagnosed	53	M	inv(16)(p13.1q22) MYH11/CBFB	FLT3, CEBPA and NPM1: NEGATIVE
6	AML	Diagnosed	65	M	Normal	FLT3 ITD (+), CEBPA (-), NPM 1 (+), c kit (-), PML RARA
7	AML	Newly Diagnosed	45	M	Normal	CEBPA, DNMT3A, FLT3, IDH1/2, KIT, KRAS, NRAS, RUNX1 TP53
8	AML	Diagnosed	38	F	t(8;21) RUNX1-RUX1T1	Mutations noted in KRAS, NF1, and TP53; Negative for IDH 1, IDH2 and FLT3, RUNX1
9	AML	Diagnosed	30	M	Normal	Intermediate Risk (Wild type NPM1 without FLT3-ITD without adverse risk genetic lesions)
10	AML	Newly Diagnosed	33	F	Normal	DNMT3A, NRAS, NPM1
11	AML	Newly Diagnosed	78	M	Normal	TP53,U2AF1,ASXL1,RUNX1, FLT3-ITD
12	AML	Newly Diagnosed	53	M	46,XY,r(3)(p26q29),del(5)(q22q35),der(7)t(7;?;3)(q22;?;p11)[17]	TP53
13	AML	Newly Diagnosed	37	F	Normal	WT1, FLT3-ITD
14	AML	Newly Diagnosed	40	M	Normal	DNMT3A, IDH2, KRAs, NRAS,NPM1
15	AML	Newly Diagnosed	72	F	Normal	DNMT3A, TET2
16	AML	Newly Diagnosed	67	M	Normal	IDH2, NPM1
17	AML	Newly Diagnosed	20	M	46,XY,inv(16)(p13.1q22.1)[23]	KIT
18	AML	Newly Diagnosed	63	M	46,XY,i(21)(q10)[20]	RUNX1, WT1
19	AML	Newly Diagnosed	63	F	Normal	NPM1, SF3B1
20	AML	Newly Diagnosed	38	F	Normal	FLT3, WT1,NPM1,

### Mice

NRG (OD-*Rag1^null^ IL2rg^null^*) mice were purchased from the Jackson Laboratory (Bar Harbor, ME) and housed in a specific pathogen-free animal facility at Loma Linda University (LLU). All mice were used at the age of 8 weeks. All experiments were performed in compliance with an Institutional Animal Care and Use Protocol approved by LLU Animal Care and Use Committee.

### Isolation of TILs from AML patient bone marrow samples

CD3+ T cells from bone marrow mononuclear cells (BMMNC) were separated by using CD3 microbeads (Miltenyi Biotech, Germany) and a MiniMACS™ Separator with an MS Column according to the manufacturer's protocol. Selected CD3+ T cells were considered AML TILs.

### *Ex-vivo* expansion of high number TILs by a traditional T cell protocol

CD3+ TILs were isolated from AML BMMNC by the pull-down through CD3 microbeads and magnetic separation. The non-CD3+ cells (feeder cells) were pre-treated with 10 µg/mL mitomycin-C for 2 h to arrest cell proliferation. CD3+ TILs and feeder cells were co-cultured at 37 °C and 5% CO_2_ in a RPMI 1640 culture medium containing 10% fetal bovine serum (FBS, HyClone), 100µg/ml penicillin/streptomycin, and Interleukin (IL-2) (1000 U/ml, Peprotech). Seeding cell density was 300 µl of 100,000 cells/ml in each well of 48-well-plates. For the maintenance of quickly expanded TILs, we performed a medium change every 2, 3 days, and split cells at the ratio of 1:4 when reaching 80 % confluent. TILs were stimulated with 30 ng/mL human anti-CD3 (OKT3, Biolegend). Around 10–14 days, we started to culture TILs in 12-well-plates or T25 flasks for expansion of large amounts before further analyses.

### *Ex-vivo* expansion of low number TILs by a modified T cell protocol

#### b/1) media with cytokines

CD3+ T cells were cultured at 37 °C and 5% CO_2_ in a RPMI 1640 culture medium containing 10% fetal bovine serum (FBS, HyClone) with penicillin/streptomycin (100 µg/ml), IL-2 (1000U/ml, Peprotech), and Dynabeads® Human T-Activator CD3/CD28 (Gibco^TM^) without feeder cells.

#### b/2) timeline of TIL expansion

*Stage 1 (Naïve TILs)***:** The seeding cell density of CD3+ TILs was around 300 µl of 20,000 cells/ml in appropriate wells of 48-well-plates. Due to the low cell density of TILs, we added fresh media at a 1:1 ratio to each well every 2 days and mixed the cells/media. Based primarily on the growth of the TILs, we performed media change every 5–7 days and split cells at the ratio 1:3.

*Stage 2 (Ready to grow):* After 7 days, IL-7 (25ng/ml, Peprotech) and IL-15 (25ng/ml, Peprotech) were added to the media along with IL-2 (1000U/ml). Every patient BMMNC sample was different; however, we preferred to raise TILs in 48-well-plates for expansion to sufficient amounts during the beginning 10–14 days instead of in large wells and flasks.

*Stage 3 (Quickly expand and differentiate into T effectors):* After 10–14 days, TILs grew very fast. We performed media change every 2 days and split quickly expanded TILs at the ratio 1:4 to 1:8. Then, we expanded TILs in multiple 48 or 24 well-plates. Dynabeads® Human T-Activator CD3/CD28 was used once for re-stimulation of TILs.

### Flow cytometry (FACS)

Expanded TILs were harvested and examined for the expression of cell surface biomarkers (CD) and intracellular proteins for T cells by multichromatic FACS. Briefly, about 1 × 10^4^ ∼10^6^ cells in 100 µl FACS buffer (PBS containing 1% FBS and 0.05% sodium azide) were stained with various fluorescence-conjugated antibodies specific for the desired cell surface proteins at 4 °C for 30 min. The surface-stained cells were then fixed and permeabilized using the appropriate reagents (e.g. the BD Pharmingen Cytofix/Cytoperm buffer) and stained with different fluorescence-conjugated antibodies specific for the desired intracellular proteins at 4 °C for 30 min in the permeabilizing buffer (e.g. the BD Perm/Wash buffer). Finally, the cells were washed twice in the permeabilizing buffer and twice in the FACS buffer before being analyzed on the BD FACSAria II. Data was analyzed using the FlowJo software (Treestar).

### Cytotoxicity assay

We performed the cytotoxicity assays by co-culturing engineered TILs with primary AML blasts from the same patient (isolated by CD33-microbeads pull-down) in 24-well plates. AML blasts from BMMNC were separated using APC anti-human CD33 antibody (Biolegend), anti-APC microbeads (Miltenyi Biotech, Germany), and a MiniMACS™ Separator with an MS Column. The ratio of autologous TILs to AML blasts were in the range of 5:1–10:1 according to a previous report [20]. After overnight incubation, cells were collected, stained, and processed for FACS assay of biomarkers including viability dyes (Invitrogen™) and CD33 according to manufacturers’ protocols. Analyses and graphs will be generated using the GraphPad Prism software to evaluate significance.

### Adoptive cell transplantation of engineered human AML cells and TILs in immune-deficient NRG mice

*Ex vivo* expanded TILs (2 × 10^6^ cells/mouse) were pre-labeled to be red fluorescent with Qtracker™ 655 (Molecular Probes) and intravenously (IV) injected into NRG mice through the tail vein. To help the engraftment, 10 mg/kg of Azacitidine was intraperitoneally injected one day before the injection. TILs-engrafted mice were sacrificed at different time points. In another experiment, AML cells (non-CD3+ cells from BMMNC) were transduced with GFP lentivirus to generate GFP+ AML cells. Fourteen days after transplantation of GFP+ AML cells (1 × 10^6^ cells/mouse), Qtracker™ 655 labeled TILs were IV injected into these AML NRG mice. The detailed protocol and plasmids for generating lentivirus and generating GFP+ AML cells can be found in our previous report [21]. On day 10 after TILs’ engraftment, mice were sacrificed for FACS analyses. Immunofluorescent histology was performed to visualize TILs and GFP+ AML cells inside of the bone marrow.

### Histology

Preparation of undecalcified frozen sections from bone tissues was performed according to our previous report [21]. Briefly, specimens were fixed in 4% paraformaldehyde, freeze-embedded with an embedding medium (SCEM), and frozen in pentane cooled with liquid nitrogen. The frozen specimen block was fixed to the cryostat and trimmed with a disposable blade. The block's surface was then covered with a pressure sensitive adhesive film (Cryofilm) and cut into 10 µm-thick frozen sections which were stored at -20 °C. The frozen sections were immunohistochemically stained and photographed for further analyses.

## Results

### Confirmation of the presence of TILs in bone marrows of AML patients

**Fig. 1:**
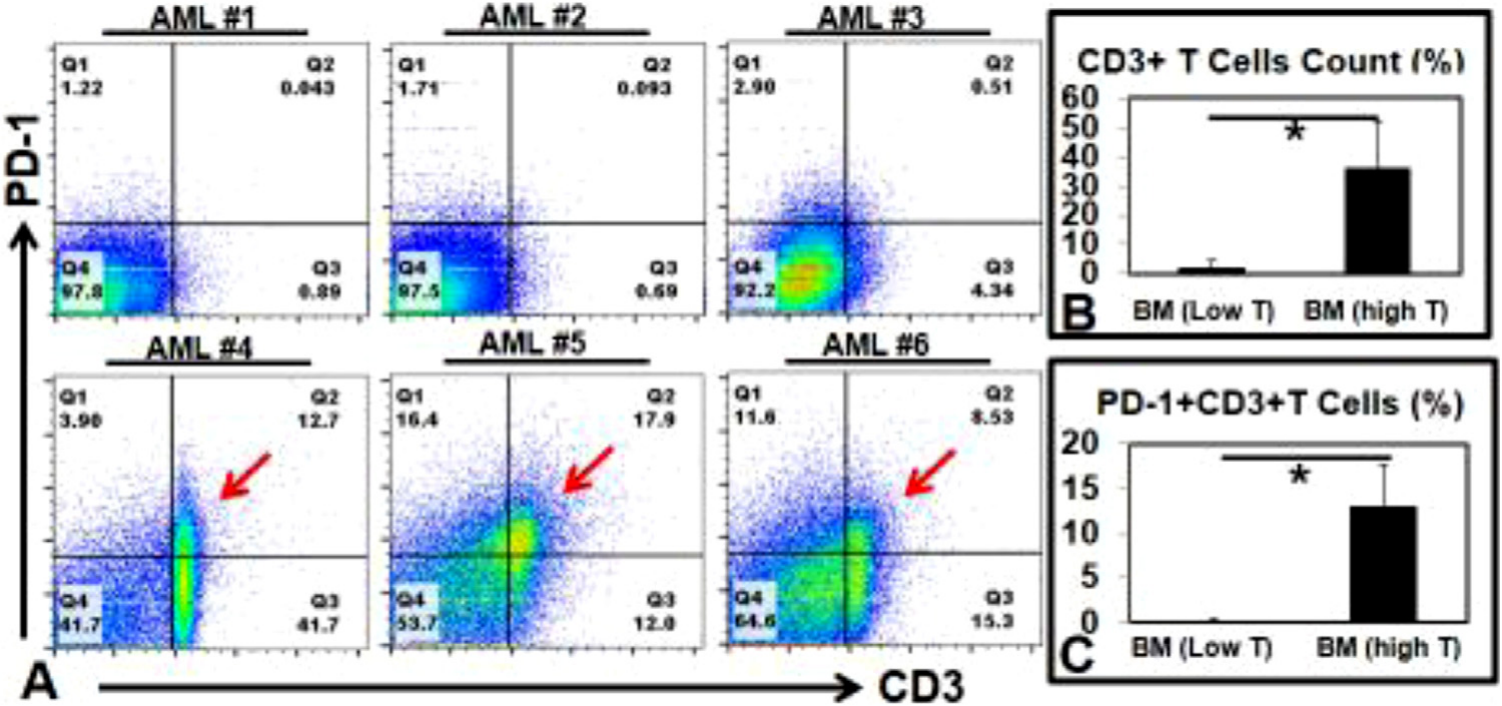
Immunophenotyping of TILs from AML patient BM samples *(A)* Representative FACS plots screening 6 AML patient BM samples; *(B)* Aggregate FACS data showing the percentage of CD3+ cells; *(C)* Aggregate FACS data showing the percentage of PD1+CD3+ cells. * *P <* 0.05.

Autologous TILs based therapies could be a novel therapeutic strategy for AML if we can (1) phenotypically identify TILs, (2) expand TILs *ex vivo* to sufficient numbers for clinical use, (3) demonstrate cytotoxic effect to autologous AML blasts and (4) bioengineer TILs to restore their Ag-specific cytotoxic functions. To this end, we firstly screened AML patient BMMNC (**Patients #1–10,** Table 1). A different degree of CD3+ T cells infiltration could be detected in all the tested samples. Overall, we observed two groups of patients with low (“Low”: upper FACS plots, Fig. 1**A; Patient #1–3, 7,** Table 1) and high (”High”: lower FACS plots, Fig. 1**A; Patient #4–6, 8–10,** Table 1) numbers of CD3+ TILs (2.3% vs 32.6%, respectively, *P* < 0.05) (Fig. 1**B**). This finding is interesting in that certain AML blast environment illicit a suppressed T cell response. Our screening data are consistent with a recent report that the CD3 TILs population are preserved in certain AML BM samples compared to health controls but about 50% of AML patients have a low T cell count in their BM. T cell-mediated cellular immunity is highly regulated by a system of checks and balances through a group of stimulatory and inhibitory proteins, including programmed death receptor 1 (PD-1) [22]. Further analyses revealed that 13% of CD3+ T cells were PD-1+ (arrows, Fig. 1**A, C**). These PD-1+ TILs are likely to have lost anti-leukemic activity against AML blasts [23], which could be restored functionally by using PD-1 inhibitors [24].

### *Ex vivo* expansion of TILs from AML BMs using a modified protocol

**Fig. 2:**
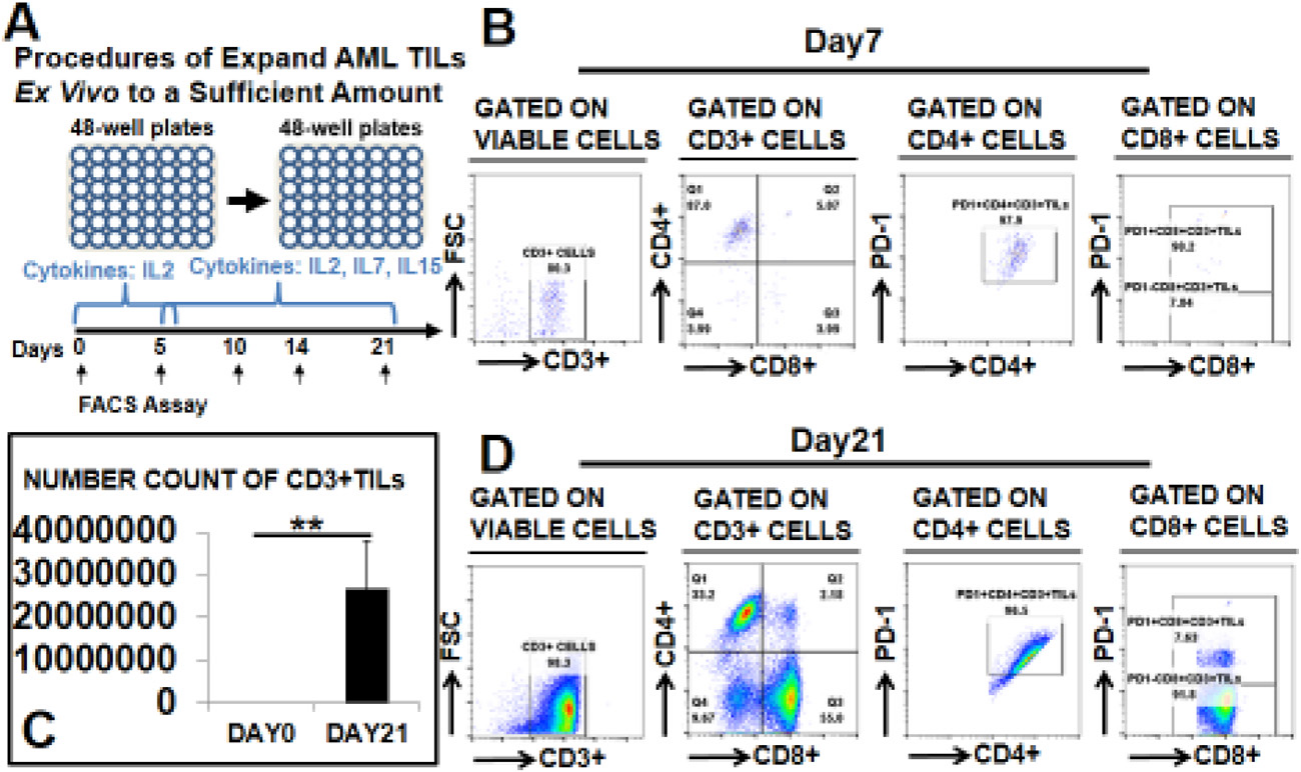
Expansion of low number CD3+TILs *ex vivo. (A)* Experimental procedures of *ex vivo* culture of isolated CD3+ TILs; *(B)* Representative FACS plots showing the CD4+ CD3+ or CD8+ CD3+ T cell subsets and their PD-1 expression on day 7; *(C)* Cumulative FACS percentage data of CD3+ T cells on day 21; *(D)* Representative FACS plots showing the CD4+ CD3+ or CD8+ CD3+ T cell subsets and their PD-1 expression on day 21; Where applicable, data are means ± SEM and were analyzed by Student *t*-test. ** *P <* 0.01.

Next, we examined the *ex vivo* expandability of AML TILs using our T cell culture system (**Supple.** Fig. 1**A**). From the “High” group, we were able to obtain 0.5 to 2 × 10^6^ CD3+ T cells/ml using CD3 microbeads. After magnetic separation, these cells (**Patient #10,** Table 1) were cultured with supporting feeder cells in RPMI-1640 supplemented with IL-2. A 4-fold increase of the CD8+CD3+ T cell population (red arrow) was obtained after the 5-day culture (30.4% on Day 5 vs 7.6% on Day 0, *P <* 0.01, **Supple.** Fig. 1**B–D**). In contrast, from the “Low” we found challenging to obtain a sufficient number of TILs. Thus, to expand these cells we utilized a modified *ex vivo* culture protocol (Fig. 2**A**). Ten vials of AML BMMNC with low T cell numbers were used in this experiment (**Patients #11–20,** Table 1). CD3 microbeads were applied to pull down the TILs from 1 ml of each BMMNC sample; 2 to 5 × 10^4^/ml CD3+ T cells were obtained and cultured with CD3/CD28 microbeads without feeder cells in RPMI-1640 supplementing them sequentially with IL-2, IL-7, and IL-15 (see experimental methods for details). At different time points, we collected cells and stained them for FACS analyses to determine their immunophenotypes. At the early stage (day 7), most CD3+ TILs were found to be CD4+ (87%, Fig. 2**B**), while also expressing PD-1+ (97.9%). At day 21, we found a three-log increase of CD3+ TIL populations (26795470 on day 21 vs 25600 on day 0, *P <* 0.01, Fig. 2**C**). Also at day 21, the percentage of CD4+ reduced to 33.2%, while CD8+ TILs increased to 55% with low expression of PD-1 (7.63%, Fig. 2**D**).

### Bioengineering expanded TILs pharmaceutically and genetically *ex vivo*

We next investigated the possibility of pharmaceutically and genetically bioengineering expanded TILs *ex vivo* (**Supple.** Fig. 2). The PD-1 pathway has received considerable attention because of its negative role during acute T cell activation and being a marker for T cell exhaustion [25]. We used nivolumab, an FDA-approved monoclonal antibody PD-1 inhibitor, to suppress PD-1 expression on TILs, as evidenced by FACS analyses (significantly reduced from 62.8% to 1.8%, **Supple.** Fig. 2**A–C**). Previously, we reported a new Vitamin D gene therapy to treat AML mice by overexpressing the CYP27B1 ectopic gene, which encodes the 1-alpha-hydroxylase to generate active Vitamin D *in situ* [21]. In this study, using a lentivirus system we also genetically engineered *ex vivo* expanded TILs, which were demonstrated to overexpress the CYP27B1 ectopic gene (arrow, **Supple.** Fig. 2**E**). In future studies, we will examine the anti-leukemia function of CYP27B1+ TILs and also explore whether TILs will be a potential cell vehicle candidate for gene therapies.

### Investigate possible explanations for difficulties with expansion of CD3+ TILs in some AML patients

**Fig. 3:**
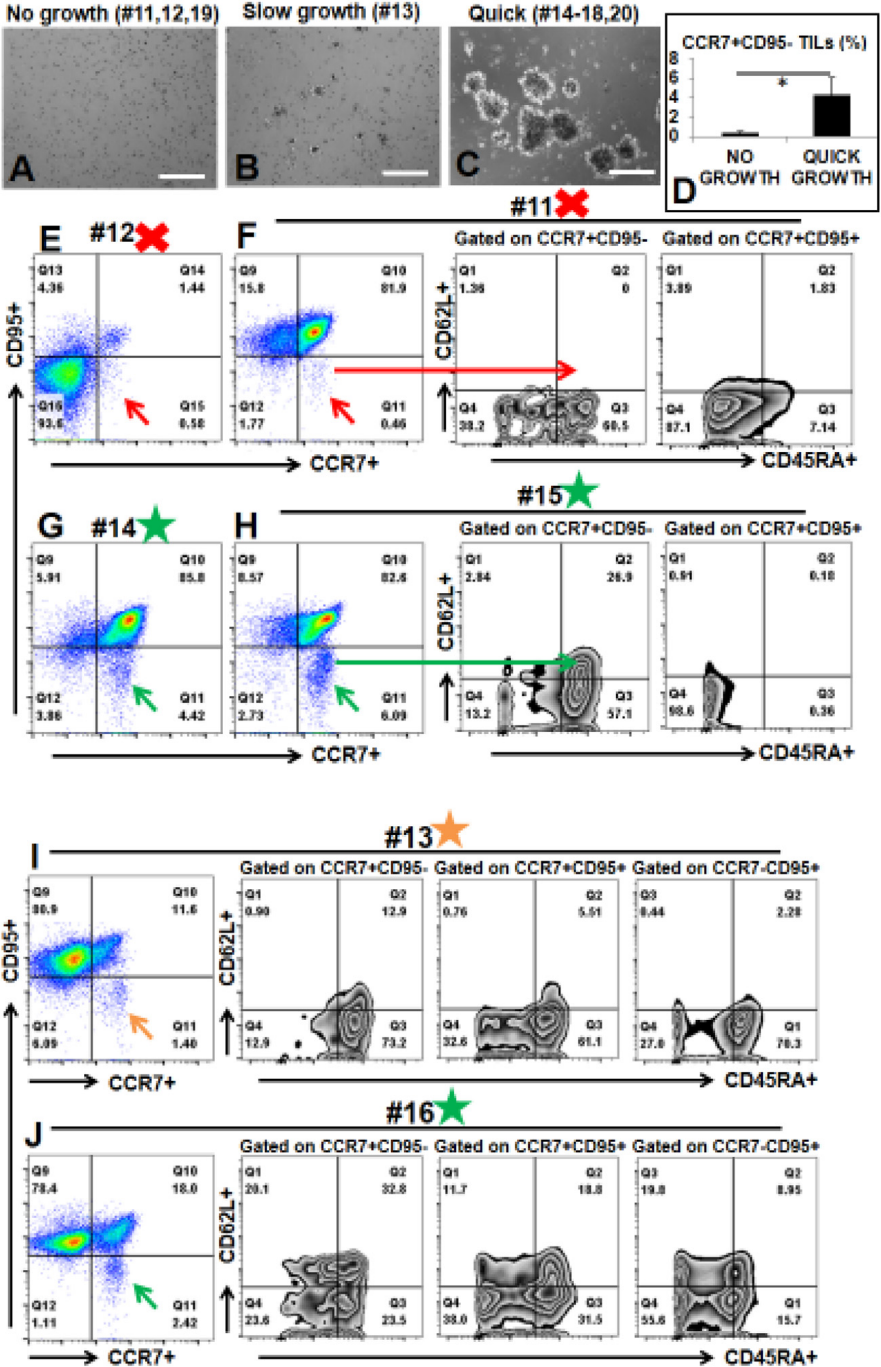
The existence of Naïve TILs in AML patient BM samples correlates to their proliferation capabilities *ex vivo (A–C)* Representative phase-bright images showing different growth of CD3+ TILs; Scale bar: 100 µm; *(D)* Cumulative FACS percentage data of CCR7+CD95-TILs between No growth and Quick growth; *(E)* Representative FACS plots of TILs with no growth from Patient #12; Red arrow indicates CCR7+CD95- cells; *(F)* Representative FACS plots of TILs with no growth from Patient #11; Red arrow indicates CCR7+CD95- cells; Long red arrow indicates CCR7+CD95- CD62L+CD45RA+ cells; *(G)* Representative FACS plots of TILs with quick growth from Patient #14; Green arrow indicates CCR7+CD95- cells; *(H)* Representative FACS plots of TILs with quick growth from Patient #15; Green arrow indicates CCR7+CD95- cells; Long green arrow indicates CCR7+CD95- CD62L+CD45RA+ cells; *(I)* Representative FACS plot of TILs with slow growth from Patient #13; Brown arrow indicates CCR7+CD95- cells; *(J)* Representative FACS plot of TILs with quick growth from Patient #16; Green arrow indicates CCR7+CD95- cells; # indicates Patient No; Red X mark indicates no growth; Brown Star mark indicates slow growth; Green Star Mark indicates quick growth (For interpretation of the references to color in this figure legend, the reader is referred to the web version of this article.)

**Fig. 4:**
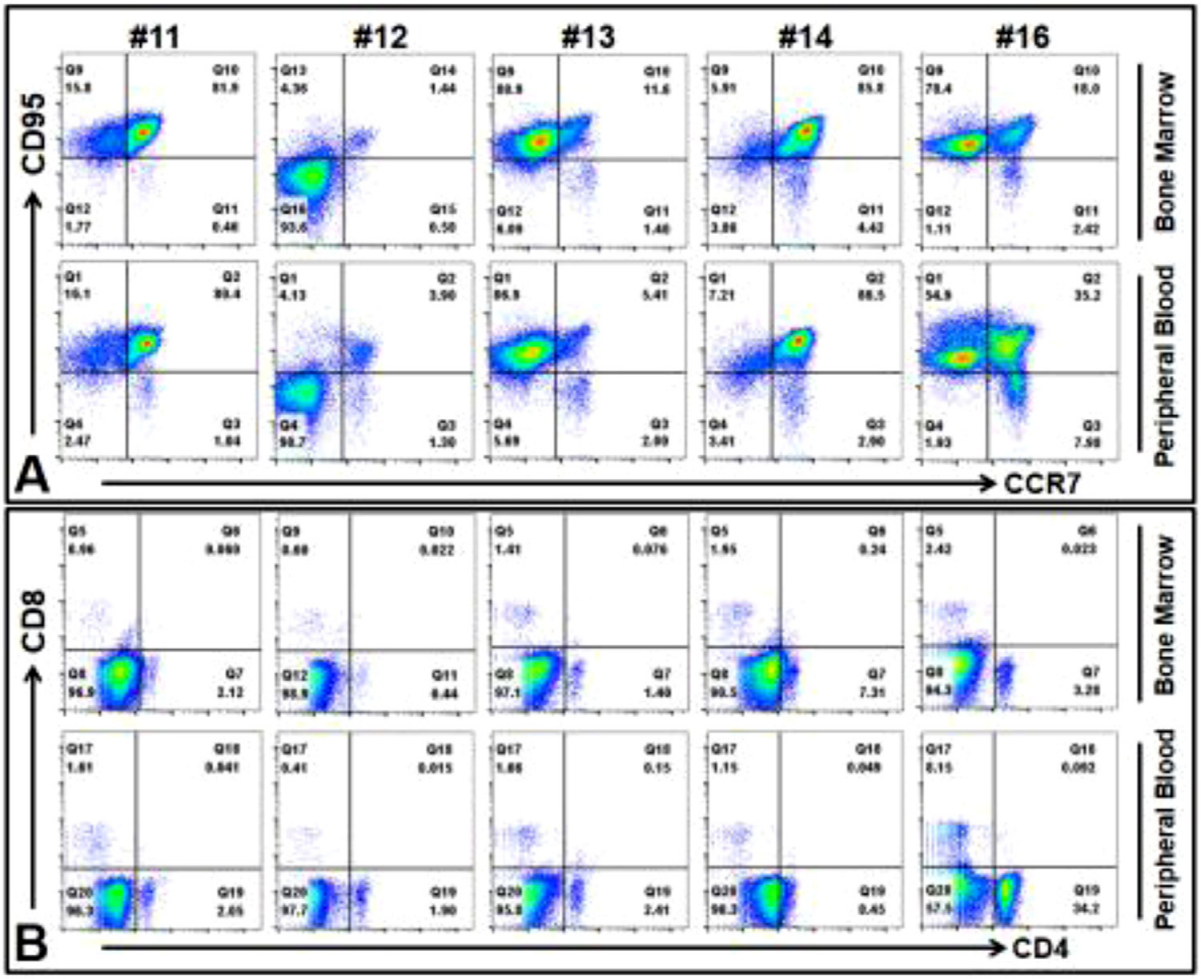
Comparison of naive T cells and differentiated T cells in bone marrow and peripheral blood of same patients *(A)* Representative FACS plots of naïve T cells in both bone marrow and peripheral blood; *(B)* Representative FACS plots of differentiated T cells in both bone marrow and peripheral blood.

During the culture of TILs from 10 AML patient samples, one consistent aspect for TIL cultures (*n =* 10) was that the proliferation status of TILs during the early stages (days 3–5) was a good predictor for whether TILs (representative images of early TIL clusters, Fig. 3**C**) could be expanded to clinical scale in later stages. We found that not every sample could generate TILs *ex vivo* (Fig. 3). After CD3 microbead pull-down, CD3+ T cells were present in these BMMNC samples (patients #11, 12, 19), but they failed to expand (Fig. 3**A**) and generate TIL clusters (Fig. 3**C**). There was another sample (patient #13) which could generate small clusters (Fig. 3**B**), but it grew relatively slow compared to those quickly expanding TILs (patients #14–18, 20, Fig. 3**C**). To investigate the mechanism underlying differential growth capabilities of TILs, we performed an immunophenotypic comparison of these AML BMMNC by using biomarkers for naïve T cells, including CD62L, CD45RA, CCR7, CD95 [26]. No significant difference of CD62L+CD45RA+ naïve TILs was found between the no/slow growth BMMNC and the quick growth BMMNC(P=0.38, **Supple.** Fig. 3). However, we found that there was a significant loss of CCR7+CD95- naïve T cell population (red arrow, Fig. 3**E, F**) in patients #11 and #12 (9.6-fold decrease, 0.45% of no growth vs 4.31% of quick growth, *P <* 0.05, Fig. 3**D**). There were clear CCR7+CD95- naïve T cell populations in patients #14, #15, and #16 (green arrow, Fig. 3**G, H, J**), part of which also expressed CD62L+CD45RA+ naïve biomarkers. When comparing the detailed immunophenotypic pattern of patient #13 (slow growth) with patient # 16 (quick growth), we found that there was a 3-fold increase of CD62L+CD45RA+ cells in patient #16 BMMNC versus patient #13 BMMNC in each compartment of CCR7+ or CD95+ subpopulations (Fig. 3**I, J**). Our data suggest that to effectively expand TILs to a sufficient amount, the CCR7+CD95- naïve T cell population in AML patient BM are needed to support the quick expansion *ex vivo*. To explore alternative sources of T cells for TILs therapy in patients with low BM TILs, we investigate whether their peripherally isolated T cells can be expanded by our novel culture system. We first found similar patterns of naïve T cells and differentiated T cells in the peripheral blood (PB) and BM samples of same patients (Fig. 4**A, B**). Next, expansion culture experiments revealed similar growth patterns between PB and BM samples including no expansion of #11 and #12 PB samples *ex vivo* (data not shown).

In all, the presence of naïve T cells in the marrow of a subgroup of AML patients was found to be critical for sufficient expansion of TILs and further treatment development.

### Functional characterization of *ex vivo* expanded TILs from AML patients using *ex vivo* cytotoxic assays and *in vivo* homing assays

**Fig. 5:**
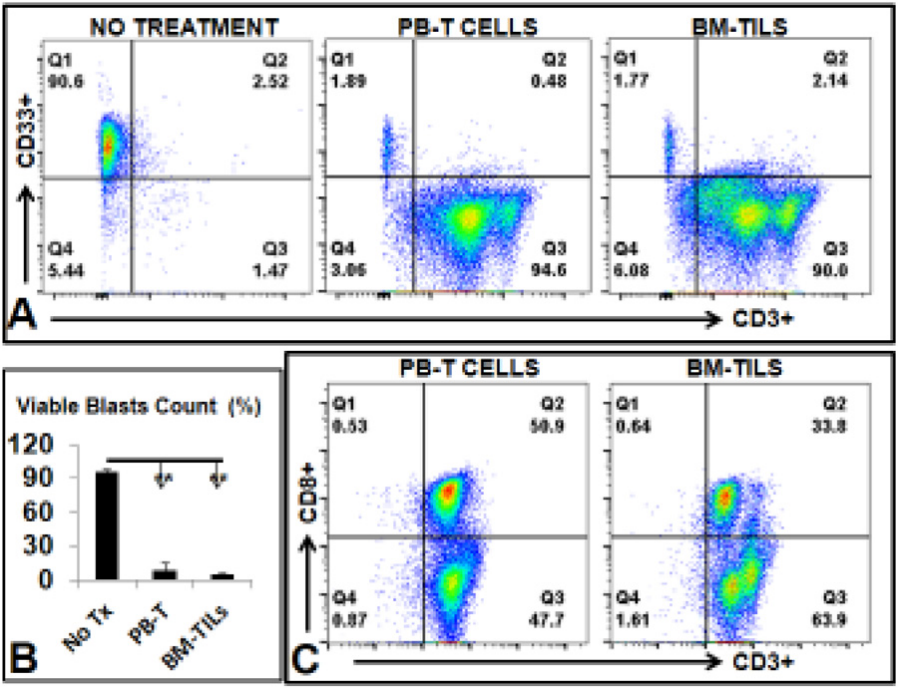
Cytotoxic Tests of #14 AML PB-T and BM-TILs *ex vivo (A)* Representative FACS plots showing the percentage of viable AML blasts in the cytotoxic test; The T cells versus blasts ratio equals 10:1. *(B)* Cumulative FACS percentage data of viable AML blasts; *(C)* Representative FACS plots showing the percentage of viable CD8 percentage; Where applicable, data are means ± SEM and were analyzed by Student *t*-test. ** *P <* 0.01, *N =* 3.

To examine the function of *ex vivo* expanded TILs, we performed cytotoxic tests. CD33, a surface biomarker, is expressed on leukemia blasts from the majority of AML patients [27]. 0^4^-10^5^ autologous CD33+ blasts with 2 × 10^5^-10^6^ isolated and *ex vivo* expanded TILs (E: T ratio 10:1). After 18 h, cells were collected and stained for FACS analysis. We observed a significant decrease of viable CD33+ blast population in TIL treatment versus the control of no treatment group (90.6% vs. 1.89%; *p <* 0.01) (Fig. 5**A, B**). TILs expanded from either PB or BM from the same patients were similarly effective against autologous blasts.

To investigate whether *ex vivo* expanded TILs will home to the BM and maintain their proliferation and functional capabilities *in vivo*, we performed a set of preliminary transplantation experiments. TILs were pre-labeled with Qtracker 655 and then intravenously injected to naïve immune-deficient mice (NRG) (*n* = 5). On day 14, mice were sacrificed and examined for the location of transplanted TILs. Transplanted TILs were found in the BMs of NRG mice, and continued to express CD3+ (**Supple.** Fig. 4**B**). We then engrafted AML blasts (GFP-labeled) in NRG (*n =* 5) mice followed by infusion of TILs (Qtracker 655 labeled) (see experimental procedures, **Suppl.** Fig. 4**A**). On day 24, mice were sacrificed for histology. TILs were found in BMs next to with GFP-labeled AML blasts (**Supple.** Fig. 4**C**).

## Discussion

In this study, we demonstrated the presence of TILs in AML patients’ bone marrow samples. We were able to characterize their phenotypic and functional features using immunophenotying and cytotoxic assay. To examine the *ex vivo* expandability of TILs for the possibility of autologous transplantation, we developed a novel *ex vivo* culture system to expand TILs from AML patient BM samples with low numbers of CD3+ T cells to clinical scales. Furthermore, we immunophenotypically determined that these TILs expressed either CCR7+CD95-/or CD62L+CD45RA+, which are makers for naive T cells [28]. We have observed that some patients have high numbers of CD3+ TILs while others have low numbers of CD3+ TILs in their BM. The presence of naïve T cells is the hallmark of expandability of T cells, even in patients with low initial CD3+ TILs. Finally, we demonstrated that TILs can cause cytotoxicity to autologous blasts *ex vivo*, can be engineered to express desirable genes, and are able to migrate to BM after being transplanted into immunodeficiency mice *in vivo*. As a preliminary *in vivo* experiment, our results provided evidence that transplantation of expanded TILs is feasible and that we could track IV injected cells to the bone marrow. These *ex vivo* expanded TILs are likely to maintain their BM homing capability, proliferation and therapeutic capabilities *in vivo*. Our current preliminary *in vivo* data also suggested that primary TILs could be engineered to overexpress a desirable gene for therapeutic purpose as we previously showed (**Supple.** Fig. 2**E**). Thus, TILs could be used a vehicle for gene therapy for autologous transplantation to treat AML. In future experiments, it would be important to compare homing, proliferating, cytotoxic and therapeutic capabilities between BM derived TILs with circulating TILs from peripheral blood (PB) *ex vivo* and *in vivo* transplantation studies. Our results suggest that BM derived TILs based cell therapies is a promising, novel therapeutic strategy for AML patients and should be further explored.

### Significance of our current study for basic research of TILs and clinical application

The complexity of AML suggests that AML patients require personalized therapies to achieve long term remission [13,29]. Previous studies have demonstrated that availability of CD3+ TILs and high percentages of CD8+ TILs *in situ* were essential in preventing disease progression or relapse, and prolonging the survival in cancer patients [30,31]. Very little is known of whether TIL have a role in the evolution and treatment-response of AML patients and whether TIL-based approach can be used to elicit a therapeutic immune response. Thus, understanding how the immune system in AML BM interacts with malignant cells and the tumor microenvironment is likely to be critical for the development of successful immunotherapeutic strategies [32]. In this study, we showed that the presence of TILs in the BM from AML patients, albeit with a variable degree. Whether the level of BM TIL infiltration has any prognostic significance, however remains to be determined.

**Fig. 6:**
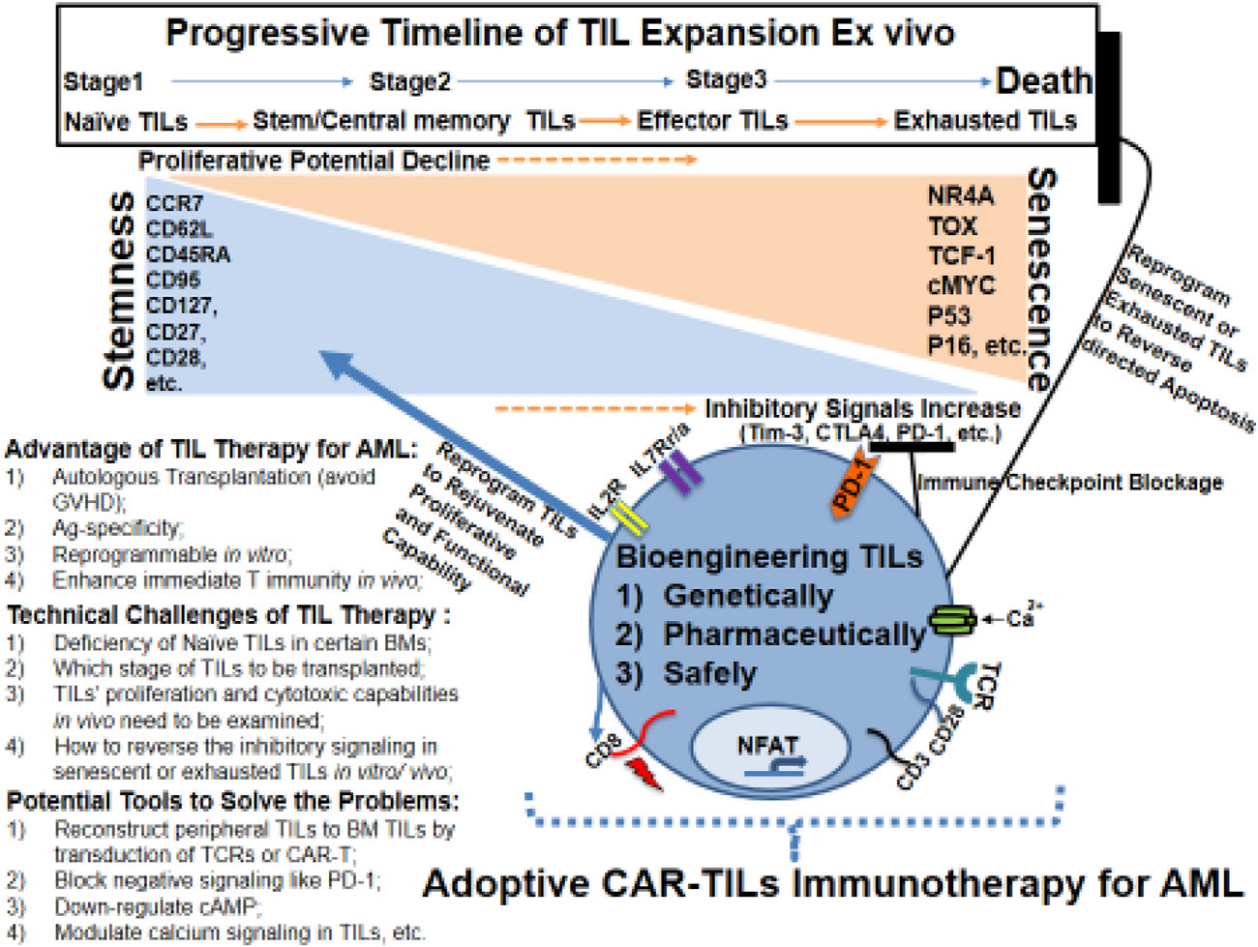
Summary Cartoon of TILs based immunotherapy for AML.

Naive T cells are immature cells, commonly characterized by the surface expression of CD62L (L-selectin) and CCR7 (C-C Chemokine receptor type 7) [33]. In addition, serial adoptive transfers of a single CD62L^+^ memory T cell, a subset of a naïve T cell, demonstrated its stemness including self-renewal and multipotent capabilities *in vivo* [34]. Herein, we report that CCR7+CD95-/or CD62L+CD45RA+ naïve T cells exist in some AML BMs, which could be isolated and expanded by our modified *ex vivo* culture system (*n =* 7/10). By using our novel TIL culture method, we could *ex vivo* expand TILs over a three log-fold, which would be useful for studying subsets of TILs and bioengineering them to fit potential clinical applications for AML (Fig. 6). Our TIL protocol has incorporated co-stimulation from anti-CD3/CD28 microbeads supplemented with cytokines i.e., IL-7 and IL-15, which have been shown to increase the viability and induce expansion naïve T cells for sustainable expansion [35]. Interestingly, no or small subfraction of CCR7+CD95-/or CD62L+CD45RA+ were found in TILs that failed to expand *ex vivo* (Patients #11, 12, 19, Table 1). These results however will need to be confirmed in a larger cohort of AML patients and to genetic abnormalities, which would reveal the underlining mechanisms.

### Remaining questions and potential engineering tools to reverse and rejuvenate exhausted TILs for clinical applications

We recognized that albeit provocative, our results raise additional questions. For example, how long the transplanted TILs will survive in AML BM and will they expand in the BM? Their efficacy *in vivo* after transplantation is unknown, although our preliminary *in vivo* transplantation experiments showed TILs homing to the BM and identifying AML cells within a short period of times. It needs also to be evaluated whether bioengineered TILs will retain their Ag-specific functions. Recently, several key transcriptional regulators (NR4A, TOX, etc.) have been discovered to induce the anergy and exhaustion of T cells and to affect the therapeutic efficacy of immunotherapies for solid tumors 36, 37, 38, 39, 40, 41. Another question is whether we could reverse the process of TIL exhaustion and enhance their proliferative and functional capabilities *in vivo* (Fig. 6). In summary, our preliminary data provided proof of principle evidence that reprogrammable TIL based immunotherapy could potentially be a new personalized immunotherapy for the treatment of AML and relapsed/refractory AML.

## CRediT authorship contribution statement

**Huynh Cao:** Conceptualization, Investigation, Data curation, Validation, Formal analysis, Writing – review & editing. **Do Hyun Kim:** Investigation, Data curation, Validation, Formal analysis, Writing – review & editing. **Ashley Howard:** Investigation, Data curation, Validation, Formal analysis, Writing – review & editing. **Hector Moz:** Investigation, Data curation, Validation, Formal analysis, Writing – review & editing. **Samiksha Wasnik:** Investigation, Data curation, Validation, Formal analysis, Writing – review & editing. **David J. Baylink:** Writing – review & editing. **Chien-Shing Chen:** Writing – review & editing. **Mark E Reeves:** Writing – review & editing. **Saied Mirshahidi:** Investigation, Data curation, Validation, Formal analysis, Writing – review & editing. **Jeffrey Xiao:** Investigation, Data curation, Validation, Formal analysis, Writing – review & editing. **Olivia Francis:** Investigation, Data curation, Validation, Formal analysis, Writing – review & editing. **Guido Marcucci:** Writing – review & editing. **Yi Xu:** Conceptualization, Investigation, Data curation, Validation, Formal analysis, Supervision, Writing – original draft, Writing – review & editing.

## Declaration of Competing Interest

The authors declare that they have no competing interests exist.

## Appendix Supplementary materials

Image, application 1
