# Supplementary material for: Ex vivo isolation, expansion and bioengineering of CCR7+CD95-/or CD62L+CD45RA+ tumor infiltrating lymphocytes from acute myeloid leukemia patients’ bone marrow
Source: Neoplasia. 2021 Nov 11;23(12):1252–60. doi: 10.1016/j.neo.2021.11.003 (PMC8603025; doi:10.1016/j.neo.2021.11.003)
Supplement: Supplementary file 1 [file mmc1.docx]

**Supplementary Figures:**


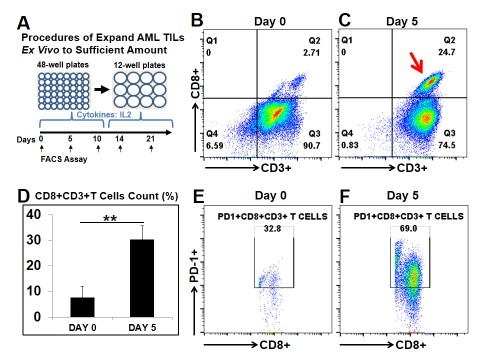


**Supplementary Figure 1: Expansion of high number CD3+TILs *ex vivo*.**

***A)*** Experimental procedures of ex vivo culture of isolated CD3+ TILs;

***B, C)*** Representative FACS plots showing the percentage data of CD3+ or CD8+ cells on day 0 and day 5;

***D)*** Cumulative FACS percentage ~~data~~ of CD8+CD3+ T cells on day 0 and day 5;

***E, F)*** Representative FACS plots showing the PD1+ CD8+ cells on day 0 and day 5;

Where applicable, data are means ± SEM and were analyzed by Student t-test. ****** P<0.01

**
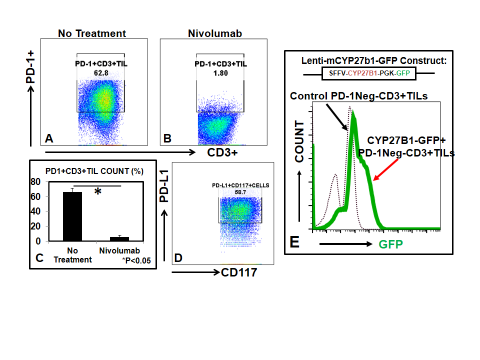
**

**Supplementary Figure 2: Bioengineering Primary AML TILs *ex vivo*.**

***A, B*)** Representative FACS plots showing the percentage data of PD-1 expression in CD3+ TILs with or without treatment of Nivolumab;

***C***) Cumulative FACS percentage data of PD-1 expression in CD3+ TILs with or without treatment of Nivolumab; N=3

***D***) Representative FACS plot of PD-L1 expression in CD117+ AML blasts;

***E***) FACS analyses of GFP expression in PD-1Negative-CYP27B1+TILs after lentiviral transduction.

Where applicable, data are means ± SEM and were analyzed by Student t-test. ***** P<0.05

**
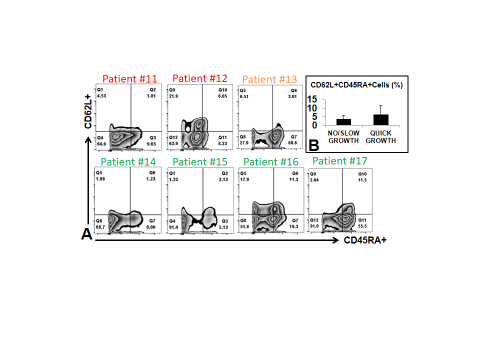
**

**Supplementary Figure 3: Comparison of CD62L+CD95+TILs in the AML Patients’ BMMNC**

***A)*** Representative FACS plots showing the percentage data of CD62L+CD45RA+TILs from different AML patient BMMNC; Red patient numbers: no growth; Brown patient numbers: slow growth; Green patient numbers: quick growth;

***B)*** Cumulative FACS percentage data of CD62L+CD45RA+TILs between No/Slow growth and Quick growth; Where applicable, data are means ± SEM and were analyzed by Student t-test.


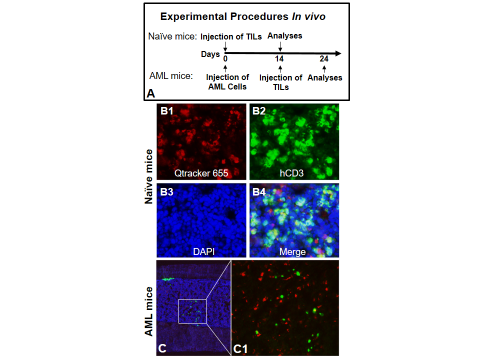


**Supple Figure 4: Transplantation of AML TILs *in vivo*.**

***A)*** Experimental procedures of transplanting TILs *in vivo*;

***B)*** Representative Immunohistochemical images showing the colocalization of Qtracker 655+ TILs (red) with CD3 expression (green) in the bone marrow of naïve mice; DAPI: blue nuclei

***C)*** Representative Immunohistochemical images showing Qtracker 655+ TILs (red) located close to AML blasts (GFP-labeled, green) in the bone marrow of AML mice on day 10;

**Supplementary** **Table 1: List of Reagents used in this study**

| **List of Reagents** | | | | |
| --- | --- | --- | --- | --- |
| **Antibody/Reagents** | **Color** | **Cat. #** | **Company** | **Species Reactivity** |
| **CD3** | PE/Cyanine7 | 300420 | Biolegend | Human |
| **CD3** | FITC | 300406 | Biolegend | Human |
| **CD4** | APC | 17-0048-41 | eBioscience | Human |
| **CD4** | PERCP | 300527 | Biolegend | Human |
| **CD8** | PerCP/Cy5.5 | 302922 | Biolegend | Human |
| **CD8** | FITC | 130-110-815 | Milteny Biotec | Human |
| **CD95** | APC | 305611 | Biolegend | Human |
| **CCR7 (CD197)** | PE | 353203 | Biolegend | Human |
| **CCR7 (CD197)** | APC | 352313 | Biolegend | Human |
| **CD62L** | FITC | 304803 | Biolegend | Human |
| **CD45RA** | PE/Cyanine7 | 304125 | Biolegend | Human |
| **PD-1 (CD279)** | FITC | 367411 | Biolegend | Human |
| **PD-1 (CD279)** | PE | 12-2799-42 | eBioscience | Human |
| **PD-L1 (CD274)** | PerCP-eFluor710 | 46-5983-42 | eBioscience | Human |
| **CD33** | APC | 303408 | Biolegend | Human |
| **Anti-APC Microbeads** |  | 130-090-855 | Milteny Biotec |  |
| **Annexin V** | FITC | 640906 | Biolegend |  |
| **Viability Dye eFluor™ 780** |  | 65-0865-14 | eBioscience |  |
| **DAPI** |  | D9542-1MG | Sigma Aldrich |  |
| **Qtracker™ 655** |  | Q25029 | Molecular Probes |  |
| **CD3 MicroBeads** |  | 130-050-101 | Milteny Biotec | Human |
| **Dynabeads® Human T-Activator CD3/CD28** |  | 11161D | Gibco | Human |
| **IL2** |  | 200-02 | Peprotech | Human |
| **IL7** |  | 200-07 | Peprotech | Human |
| **IL15** |  | 200-15 | Peprotech | Human |
| **KAWAMOTO Film Kit** |  |  | Section-Lab Co.ltd, Japan |  |
| **Azacitidine (AZA)** |  |  | Celgene |  |
| **Nivolumab (Opdivo)** |  |  | Bristol-Myers Squibb |  |
